# Straw chemistry overrides microbial and environmental controls on straw decomposition in high-latitude mollisols

**DOI:** 10.3389/fmicb.2026.1859074

**Published:** 2026-06-23

**Authors:** Ya Han, Xianghai Meng, Heng Jiang, Longkai Yue, Ziyi Wang, Qingqing Chen, Shuihong Yao

**Affiliations:** 1 Hebei Province Key Laboratory of Sustained Utilization and Development of Water Resources, Hebei GEO University, Shijiazhuang, China; 2 Key Lab of Crop Drought Tolerance Research of Hebei Province, Institute of Dry Farming, Hebei Academy of Agriculture and Forestry Sciences, Hengshui, China; 3 National-Regional Joint Engineering Research Center for Soil Pollution Control and Remediation in South China, Institute of Eco-Environmental and Soil Sciences, Guangdong Academy of Sciences, Guangzhou, China; 4 Information Center of Ministry of Ecology and Environment, Beijing, China; 5 State Key Laboratory of Efficient Utilization of Arable Land in China, the Institute of Agricultural Resources and Regional Planning, Chinese Academy of Agricultural Sciences, Beijing, China

**Keywords:** environmental factor, maize straw, microbial communities (PLFAs), straw chemical composition, straw decomposition

## Abstract

**Introduction:**

Straw returning represents a widely adopted strategy for soil organic carbon (SOC) sequestration in agricultural ecosystems, yet its efficacy is affected by straw chemistry and edaphic conditions. However, the mechanisms regulating straw decomposition in high-latitude mollisol regions remain elusive.

**Methods:**

We carried out a 17-month field experiment using the litterbag method with bags buried at 15 and 30 cm depths across two sites with different accumulated temperatures (Nenjiang and Harbin) to examine maize straw decomposition dynamics and the relationships between straw mass remaining and factors such as soil environment, soil properties, soil microbial communities, and straw chemistry. Solid-state ^13^C nuclear magnetic resonance (NMR) spectroscopy was used to characterize straw chemical composition, and phospholipid fatty acid (PLFA) analysis was employed to determine microbial community composition.

**Results and discussion:**

Maize straw mass remaining was governed primarily by site and decomposition time, with limited influence of soil depth. During decomposition, O-alkyl C (predominantly carbohydrates) continuously decreased, while Alkyl C and Aryl C (mainly lignin derivatives) gradually became enriched. The decomposition degree at the Harbin site was higher than that at Nenjiang. Bacterial and fungal communities were primarily governed by straw nitrogen, C/N ratio, and O-alkyl C, whereas both Gram-negative (G^+^) and Gram-positive (G^−^) bacteria responded to tensile strength (TS), straw nitrogen, and soil moisture (SM). Actinomycetes exhibited distinct associations with soil total nitrogen (TN), mean weight diameter (MWD), SM, TS, and straw chemistry (Carbonyl C). PLS-PM revealed that straw chemistry was strongly associated with decomposition dynamics. The soil environment, soil properties, and microorganisms jointly influence straw chemistry, thereby affecting its decomposition. These outcomes reveal the multi-factor control of straw decomposition and provide guidance for straw returning strategies in farmland ecosystems in cold regions.

## 1 Introduction

Straw returning is a sustainable practice in agroecosystems that can increase soil organic carbon (SOC) and nutrient availability, improve soil structure, and stimulate soil microbial activity while altering microbial community composition (Lal, 2004; Yang et al., 2025). Straw decomposition constitutes a fundamental mechanism for soil quality improvement; however, its efficiency is governed by complex interactions among climatic conditions, straw chemistry, soil properties and the structure and function of soil microbial communities (Wang et al., 2012; Bao et al., 2020; Shamshitov et al., 2025; Zhang et al., 2025a). While climatic conditions exert the predominant control at broad scales (Wall et al., 2008), straw chemistry operates as the primary driver consistently across spatial hierarchies, from global to local extents (Cornwell et al., 2008; Bao et al., 2020; Yuan et al., 2025). At local scales, microsite-specific soil conditions, governed by litter position, induce substantial variation in crop residue decomposition rates (Kanal, 1995; Erdenebileg et al., 2023; Yu et al., 2024). Nevertheless, the decomposition characteristics and controlling factors of maize straw in cold regions remain inadequately understood.

Straw decomposition is governed by soil environment, encompassing soil temperature and moisture regimes dictated by site characteristics and burial depth (Qu et al., 2021). The temperature sensitivity of straw decomposition is moisture-dependent, with elevated temperatures accelerating mineralization within the optimal soil moisture window (60–80% water holding capacity) (Abro et al., 2011; Huang et al., 2025). Depth-dependent variations in soil microclimate drive differential straw decomposition rates. Surface-retained straw exhibits retarded decomposition rates owing to moisture limitation via evaporative loss, whereas incorporated residues maintain favorable microclimates that accelerate decomposition (Yu et al., 2025). Soil microbial communities serve as primary mediators of straw decomposition, with fresh substrate inputs driving successional dynamics of distinct decomposer communities (Creamer et al., 2015; Zhang et al., 2025a). Decomposition typically proceeds via a bacterial-to-fungal succession, whereby r-strategist bacteria rapidly exploit labile compounds (e.g., sugars, proteins) initially, whereas K-selected fungi mediate slower mineralization of recalcitrant polymers in subsequent stages (Yang et al., 2023). Notably, emerging evidence indicates that certain Gram-positive taxa, particularly Actinobacteria, can concurrently access lignin- and polyphenol-rich polymers during early decomposition phases (He et al., 2019). Furthermore, another important factor influencing straw decomposition is straw chemistry (e.g., carbon to nitrogen ratios and/or functional groups). Straw chemistry drives microbial community assembly and taxonomic selection, in turn mediating decomposition rates (Bao et al., 2020). Straw chemistry significantly influences soil aggregation dynamics. Carbohydrate-derived moieties (O-alkyl C and Anomeric C) enhance aggregate water stability, whereas aromatic constituents exert opposing effects (Sarker et al., 2018). Recent studies have also shown that environmental physical fields (e.g., electric field, temperature gradient) can influence soil microbial activity and the decomposition of organic matter via altering microbial energy acquisition and community assembly (Zhou et al., 2026) For instance, electric fields promote hydrocarbon-degrading microbial growth, enhance organic pollutant degradation, and boost plant performance and nutrient uptake in contaminated soils (Yuan et al., 2024). These emerging findings highlight that, beyond conventional biotic and abiotic factors, physical field effects may exert important controls on straw decomposition. Despite recognition that soil conditions, microbial community structure, and straw chemistry collectively regulate straw decomposition, the underlying mechanisms remain obscure.

Mollisols are highly fertile and among the most suitable soils for crop production due to their high organic matter content (Tan et al., 2026). However, intensive cultivation has led to a continuous decline in SOC content in high-latitude Mollisols, posing a substantial threat to regional food security and soil ecological functions (Dai et al., 2024). Under this context, straw returning to the field is imperative for maintaining soil fertility, yet its effectiveness is intrinsically governed by decomposition kinetics, which are modulated by multifaceted biotic and abiotic factors. Despite the recognized importance of these factors, long-term field investigations that simultaneously track straw chemical transformation, soil microclimate dynamics, and microbial community succession in high-latitude Mollisols remain scarce. Most previous studies have either focused on single-factor laboratory incubations or short-term field observations, leaving the interactive mechanisms among straw chemistry, soil environment, and microbial communities unresolved under realistic field conditions.

Therefore, we performed a 17-month field study in the Chinese high-latitude Mollisol region to investigate maize straw decomposition across two burial depths and temperature gradients. This experimental design is particularly valuable because it mimics the two dominant straw-returning practices (surface retention vs. deep incorporation) while resolving the temperature-sensitivity of decomposition under natural climate gradients. By integrating solid-state ^13^C NMR spectroscopy and phospholipid fatty acid (PLFA) analysis, this study offers a unique opportunity to link straw chemical evolution with microbial community assembly at temporal dynamics for this region. Understanding the microbial and physicochemical mechanisms underlying straw return is critical for designing site-specific management practices that enhance soil carbon sequestration, promote aggregate stability, and preserve the long-term agroecosystem productivity of cold-region black soils. We hypothesized that: (1) straw chemical transformation and microbial community succession differ significantly between the two sites and depths; (2) microbial community assembly is jointly shaped by soil properties, soil environment, and straw chemistry; and (3) straw decomposition rates are primarily constrained by microbial community more than by soil environment, soil properties or straw chemistry.

## 2 Materials and methods

### 2.1 Site description

Our study was conducted at two sites in Heilongjiang Province, Northeast China: the Campus of Nenjiang Agricultural Sciences (Nenjiang, 49°17′N, 125°23′E) and the Minzhu Campus of Heilongjiang Academy of Agricultural Sciences (Harbin, 45°51′N, 126°47′E). The study region has a temperate continental monsoon climate. During the experiment (from 25 May 2015 to 25 May 2016), the total precipitation was 679 mm at Nenjiang and 848 mm at Harbin. The average daily soil temperatures at Nenjiang and Harbin were 8.3 °C and 11.0 °C at the 15 cm depth, and 7.6 °C and 10.9 °C at the 30 cm depth, respectively. According to the USDA Soil Taxonomy (Soil Survey Staff, 2010), soils at both sites are classified as Mollisols. However, the two soils differed in several physicochemical properties (Supplementary Table S1): the Nenjiang soil had a lower pH (6.0 vs. 6.9) but higher total nitrogen (1.8 vs. 1.5 g kg^−1^), available phosphorus (67.2 vs. 43.2 mg kg^−1^), and available potassium (307.8 vs. 152.4 mg kg^−1^), whereas the Harbin soil had higher total potassium (24.8 vs. 20.6 g kg^−1^) and silt content (43% vs. 38%). The experimental regions were chosen for their dominant cropping system: monocropping of maize (*Zea mays. L.*). The physical and chemical properties of the topsoil (0–20 cm) before the initiation of the experiment are summarized in Supplementary Table S1.

### 2.2 Experiment setup and sampling

Mature maize straw used in the experiment at both sites was collected from the Hailun Agroecological Experiment Station in October 2014. After air-drying to uniform moisture content, the straw was cut into 2–5 cm fragments. Its initial properties were as follows: 105.0 g kg^−1^ moisture, 412.7 g kg^−1^ total carbon (C), 6.1 g kg^−1^ total nitrogen (N), 2.3 g kg^−1^ total phosphorus (P), and 6.2 g kg^−1^ total potassium (K). Additionally, 2.5 g of maize straw was thoroughly mixed with 250.0 g of fresh soil, and the homogeneous mixture was loaded into nylon litterbags with dimensions of 25 cm × 10 cm and a mesh size of 74 μm. This mesh size allowed the exchange of water and nutrients and permitted microbial colonization, but excluded large soil fauna (e.g., earthworms and macroarthropods), thereby restricting the decomposition process to primarily microbially mediated pathways. These bags were designed to maintain a uniform volume (5 cm in height, 8.0 cm in diameter) and field bulk density of 1.0–1.1 g cm^−3^ (Han et al., 2020). A total of 36 litterbags were buried at each site on May 25, 2015, with half placed at a 15 cm soil depth and the other half at 30 cm. Litterbags containing the straw-soil mixture were retrieved sequentially after 1, 2, 3, 4, 12, and 17 months of incubation in the field. At each sampling time point, six litterbags were collected from the two soil depths (15 cm and 30 cm) at each study site, yielding three replicate litterbags per soil depth per time point. All collected litterbags were transported to the laboratory on the same day and stored under refrigerated at 4 °C. Straw residues were separated from the litterbags and gently rinsed with deionized water to remove adsorbed soil particles. They were then dried at 50 °C until constant weight, with their remaining dry weight recorded. Subsequently, the residues were ground into a fine powder for chemical analysis. The straw decomposition rate constant (*k*) was calculated using the formula (Wang et al., 2012; Han et al., 2020).


$$k=-\frac{1}{t}\ln\frac{Y_{t}-Y_{0}}{a}$$

where *Y_t_* represents the relative remaining mass of straw at time *t*; *Y_0_* is the asymptotic remaining mass percentage as *t* → ∞; a is the maximum mass remaining of straw residues; *k* is the decomposition rate constant.

The soil samples were divided into two subsamples: one set was air-dried for subsequent analysis of soil physicochemical properties, and the other was stored at −80 °C prior to phospholipid fatty acid (PLFA) extraction.

### 2.3 Chemical analysis of maize residues in litterbags

Total C and N in the ground straw were quantified with a Vario MACRO cube elemental analyzer, and the straw C/N ratio was calculated from the respective totals.

Solid-state ^13^C nuclear magnetic resonance (NMR) spectroscopy was employed to characterize the major C functional groups. Experiments, including ^13^C cross polarization with total sideband suppression (CP/TOSS), CP/TOSS with dipolar dephasing, ^13^C chemical shift anisotropy (CSA) filter, and ^13^C CSA filter plus dipolar dephasing, were performed on a Bruker AVANCE-III 400 spectrometer (100 MHz for ^13^C) using a 4 mm ZrO₂ rotor under 5 kHz magic-angle spinning (MAS) (Mao et al., 2017). The NMR spectra were referenced to the glycine carbonyl signal at 176.4 ppm and processed using Bruker TopSpin 2.1 software. Carbon functional groups were categorized into six classes based on chemical shift ranges (Solomon et al., 2007; Wang et al., 2012), with assignments as follows: Alkyl C (0–44 ppm), N-alkyl/methoxyl C (44–68 ppm), O-alkyl C (68–94 ppm), Anomeric C (94–113 ppm), Aryl C (113–162 ppm), and Carbonyl C (162–220 ppm). Supplementary Table S2 presents the standardized classification of organic functional groups in maize straw. The Alkyl C-to-O-alkyl C ratio and aromaticity index were employed as critical parameters to assess the decomposition extent of the straw (Solomon et al., 2007; Zak et al., 2017; An et al., 2021; Chen et al., 2024).

### 2.4 Soil physical and chemical analysis in litterbags

Soil temperature (ST) was logged *in situ* at 15 and 30 cm depths beside the litterbag with an L-93-3 probe (China). Gravimetric soil moisture (SM) was determined on 105 °C oven-dried samples. Soil organic C (SOC) and total N (TN) contents were measured by an element analyzer (Vario MACRO Cube, Germany).

Soil aggregate stability was expressed as the mean weight diameter (MWD). Air-dried soil (<8 mm) was pre-conditioned at 40 °C for 24 h. 20 g subsamples were gently slaked in deionized water on a 2 mm sieve for 5 min, then oscillated 50 times within 2 min. The procedure was repeated sequentially on 0.25 mm and 0.053 mm sieves. Fractions retained were dried at 40 °C overnight and classified as: large macro-aggregates (>2 mm), meso-aggregates (2–0.25 mm), micro-aggregates (0.25–0.053 mm), and silt + clay (<0.053 mm). MWD was calculated as the formula (van Bavel, 1949):


$$\mathrm{MWD}=\sum_{1}^{n+1}\frac{r_{i-1}+r_{i}}{2}\times m_{i}$$

where *r_i_* is the aperture of the *i*^th^ sieve (*r*_0_ = *r*_1_ and *r*_n_ = *r*_n + 1_), *m*_i_ is the mass proportion retained, and *n* is the number of sieves.

Tensile strength (TS) of individual aggregates (2–5 mm) was determined by the crushing method of Dexter and Kroesbergen (1985). Each aggregate was compressed between parallel plates at 1.6 mm·min^−1^ until failure; the peak force (*F*, N) was recorded. TS was calculated as:


$$\mathrm{TS}=0.576\times \frac{F}{D^{2}}$$

where *D* (m) is the effective diameter obtained from:


$$D=D_{0}\times {\left(\frac{M}{M_{0}}\right)}^{\;\frac{1}{3}}$$

where *D*_0_ = (*d*_1_ + *d*_2_)/2, *d*_1_和*d*_2_ are the diameters of upper and lower sieves, respectively; *M* is the mass of one aggregate, and *M*_0_ is the mean mass of all the aggregates.

### 2.5 Soil microbial community composition

Phospholipid fatty acids (PLFAs) were extracted from 2.5 g freeze-dried soil following the method (Bossio et al., 1998). Briefly, lipids were extracted with 9.0 mL chloroform-methanol-citrate buffer solvent (1:2:0.8, v: v: v) for 2 h, centrifuged at 3000 rpm for 10 min, and the supernatant was transferred to a new tube. The soil was extracted by 7.0 mL chloroform-methanol-citrate buffer again. The supernatant from the second extraction was added to the first tube. Then 5.0 mL chloroform and 5.0 mL citrate buffer were added, the chloroform layer was separated and dried by a rotary evaporator at 35 °C. Phospholipids were eluted from neutral and glycolipids with methanol through solid phase extraction columns (Agilent Bond Elut SI), separated into neutral lipids, glycolipids and phospholipids by sequential elution with chloroform, acetone and methanol, and dried. The extracted PLFAs were hydrolysed and methylation by using mixture of methanol-toluene mixture (1:1, v: v) and methanolic KOH solution, and heating 37 °C for 15 min, then 2-mL H_2_O and 0.3-mL 1.0 M acetic acid were added. The resulting PLFA methyl esters were dissolved by hexane and analyzed using Gas Chromatograph Agilent 6,850 (Agilent Technologies, CA, United States) with a flame ionization detection. Peaks were quantified using the MIDI peak identification software (Version 6.2, MIDI, Inc., Newark, DE, United States) and methyl nonadecanoate (19:0) was used as an internal standard.

The phospholipid fatty acids (PLFAs) were categorized into five microbial functional groups: (1) General bacteria (GB): 14:0, 16:0, 17:0, 18:0 (Zelles, 1999; Zhang et al., 2013); (2) Gram-negative (G^−^) bacteria: 15:1ω6c, 16:0 N alcohol, 16:1 2OH, 16:1ω5c, 16:1ω7c, 16:1ω11c, i16:1G, 17:1ω8c, a17:1ω9c, a17:1B, cy17:0, 18:1ω5c, 18:1ω7c, 19:1ω6c, cy19:0, 20:1ω9c (Hamman et al., 2007; Hong et al., 2015); (3) Gram-positive (G^+^) bacteria: i14:0, i15:0, a15:0, i16:0, i17:0, a17:0, i18:0 (Creamer et al., 2015); Actinomycetes (Act): 10Me16:0, 10Me17:0, 10Me18:0, 10Me19:0 (Frostegård et al., 1993); (4) Fungi (F): 18:1ω9c, 18:2ω6,9c, 18:3ω6c(6,9,12), 20:2ω6,9c (Cong et al., 2015; Myers et al., 2001; Olsson et al., 1999); The abundance of individual PLFAs was quantified as the absolute carbon content in soil (nmol g^−1^). The G^+^/G^−^ ratio is a dynamic indicator of carbon availability for soil microbial community during straw residue decomposition (Fanin et al., 2019).

### 2.6 Statistics

Statistical analyses were performed using SPSS 26.0. *t*-tests were applied to determine significant differences (*p* < 0.05) in soil basic properties and straw decomposition rates between treatments. Three-way analysis of variance (ANOVA) was used to assess the effects of experimental site (S), soil depth (D), and decomposition time (T) on soil physicochemical properties and microbial group abundance. Tukey HSD test was employed for post-hoc multiple comparisons. Redundancy analysis (RDA) was performed to explore the relationships between microbial community composition (35 PLFAs) and environmental variables. The RDA axes represent linear combinations of the environmental variables that optimally account for the variation in PLFA profiles. Mantel tests based on Bray-Curtis distance matrices were employed to examine linkages between major soil microbial groups and soil environmental factors, soil physicochemical properties and straw chemistry. Partial Least Squares Path Modeling (PLS-PM) was conducted using the plspm package in R software (version 4.2.0) to quantify the direct and indirect impacts of soil environmental factors, soil properties, microbial groups, and straw chemistry on straw mass remaining. The model goodness-of-fit (GOF = 0.54) was used to evaluate the overall model quality. The analysis included 48 samples and 20 observed variables, yielding a sample-to-variable ratio of 2.4:1, which satisfies the minimum requirement for stable model estimation.

## 3 Results

### 3.1 Dynamics of maize straw decomposition and chemical composition

**Figure 1:**
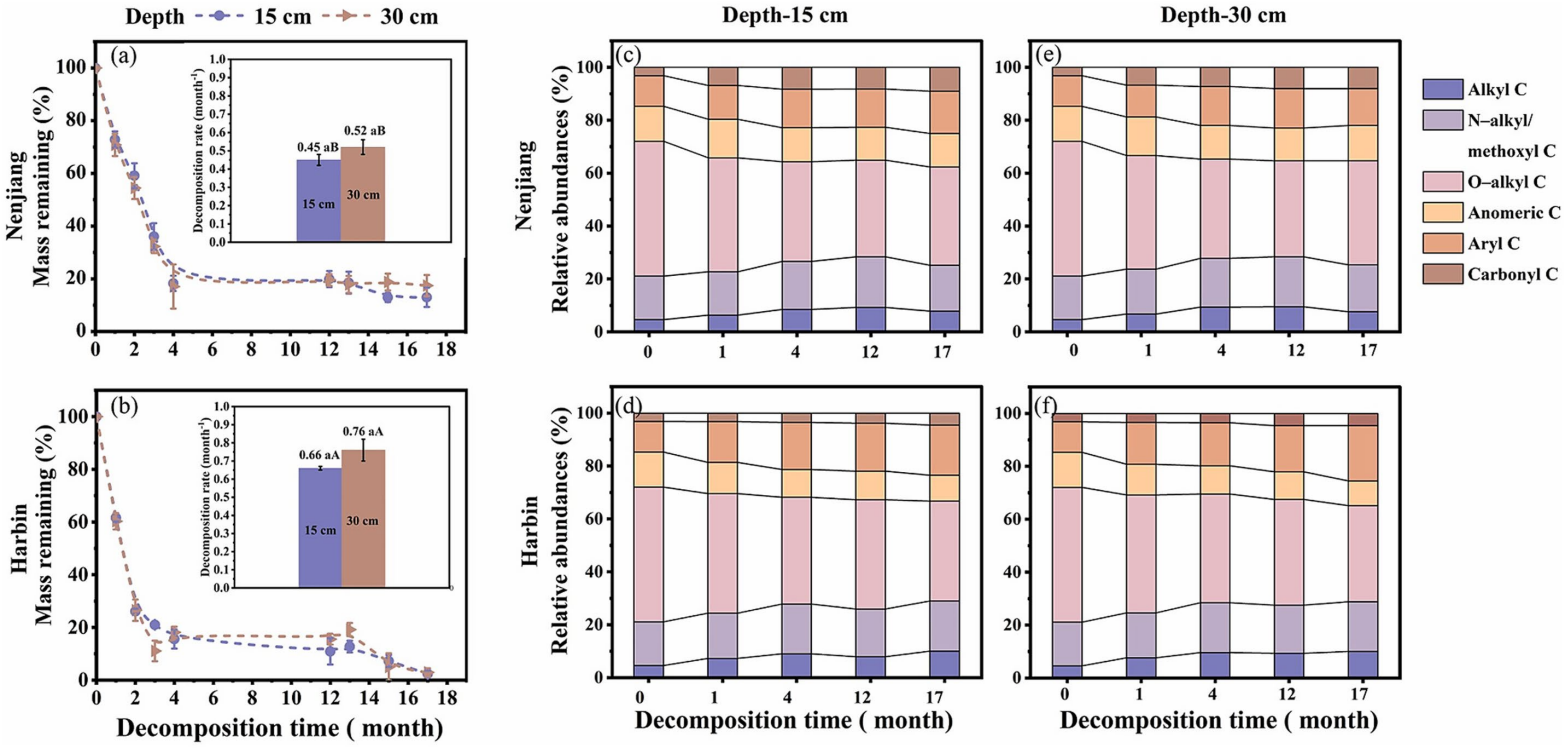
Mass remaining of straw residues **(a,b)** and relative abundances of six functional groups determined by the ^13^C-NMR technique **(c–f)** at two soil burial depths (15 and 30 cm) at the Nenjiang and Harbin sites across successive stages of decomposition. Lowercase letters over the columns **(a,b)** represent significant differences (*p* < 0.05) between burial depths, whereas uppercase letters indicate significant differences (*p* < 0.05) between sites at the same burial depth.

The decomposition of maize straw exhibited a consistent trend across all treatments, with the trend of rapid decomposition in early stages and slow decomposition in later stages (Figures 1a,b; Supplementary Table S3), with decomposition time (T) being the dominant factor affecting straw mass remaining (*p* < 0.001). Specifically, significant mass loss occurred during the early decomposition period (0–4 months), with mass remaining dramatically decreasing from 100% to approximately 17.07–18.27% at Nenjiang and 15.60–18.13% at Harbin. This was followed by a stabilized phase (4–12 months) where decomposition moderated substantially, ultimately approaching dynamic equilibrium (12.97–17.43% at Nenjiang and 2.41–2.95% at Harbin for 17 months). Site (S) had a significant effect on straw decomposition (*p* < 0.001). During the decomposition period, the mass remaining of straw at Harbin site was higher than that at Nenjiang site (19.54% vs. 27.72%, Supplementary Table S3), indicating faster decomposition of maize straw in the warm temperate continental monsoon climate of Harbin. In contrast, soil depth (D) had no significant effect on straw mass remaining (*p* = 0.618), with the average mass remaining at 15 cm (26.81%) and 30 cm (27.72%) being relatively close. Additionally, the interaction between site and time (S × T) did not reach a significant level (*p* = 0.123), suggesting that the temporal pattern of straw decomposition was consistent across the two sites. Maize straw decomposition was characterized by initial rapid carbon (C) loss and nitrogen (N) immobilization, with the C/N ratio declining from 68.17 to approximately 20.69–30.67 during the 17-month decomposition period (Supplementary Figure S1). Site significantly regulated N dynamics and late-stage C/N, whereas soil depth exerted minimal effects.

**Figure 2:**
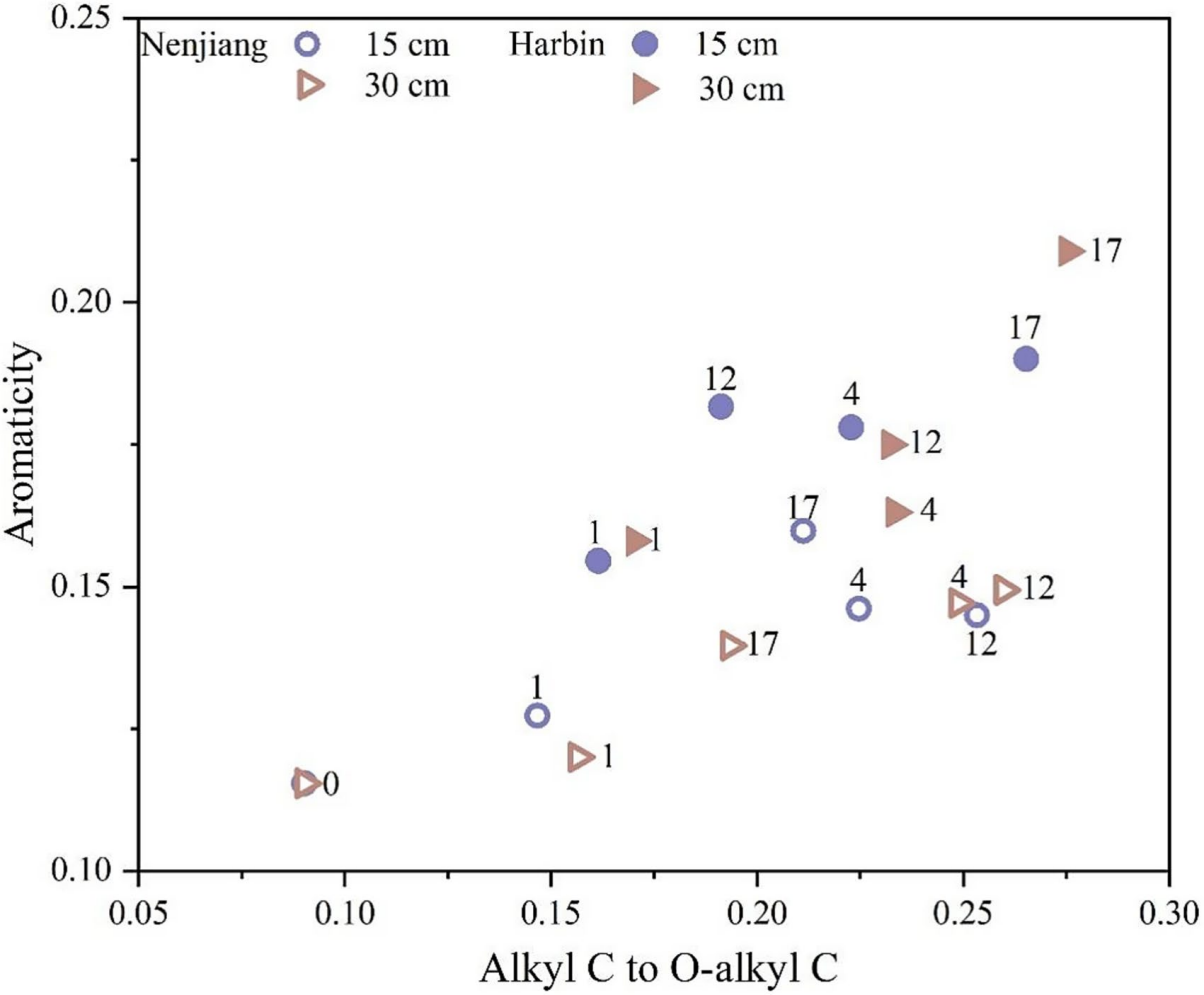
Variations in aromaticity and Alkyl C to O-alkyl C ratios of straw residues along soil burial depths (15 and 30 cm) at the Nenjiang and Harbin sites during different stages of decomposition.

Based on ^13^C-NMR analysis, six organic functional groups (alkyl C, N-alkyl/methoxyl C, O-alkyl C, anomeric C, aryl C, and carbonyl C) in maize straw showed distinct dynamic changes during decomposition, and the variation characteristics differed among sites and soil depths (Figures 1c–f; Supplementary Table S2). O-alkyl C, the predominant group in fresh straw (51.0%), exhibited a sustained decline during decomposition, signifying continuous carbohydrate and cellulose degradation. At the Harbin site, its relative abundance decreased to 36.3–37.7% at 17 months, while at the Nenjiang site, it dropped to 37.16–39.34%. Conversely, alkyl C and aryl C demonstrated increasing trends, reflecting aliphatic compound accumulation and lignin-derived aromatic compound enrichment, respectively. Carbonyl C also increased slightly, with higher levels at Nenjiang (Figures 1c–f). Although the Alkyl C/O-alkyl C ratio surged at both sites, Harbin maintained consistently greater values, indicating more advanced decomposition stages at this location. Additionally, aromaticity increased from 0.12 to 0.14–0.21 and higher at Harbin (Figure 2). These findings emphasized the complex interplay of site-specific factors in straw decomposition.

### 3.2 Dynamics of microbial community composition

**Figure 3:**
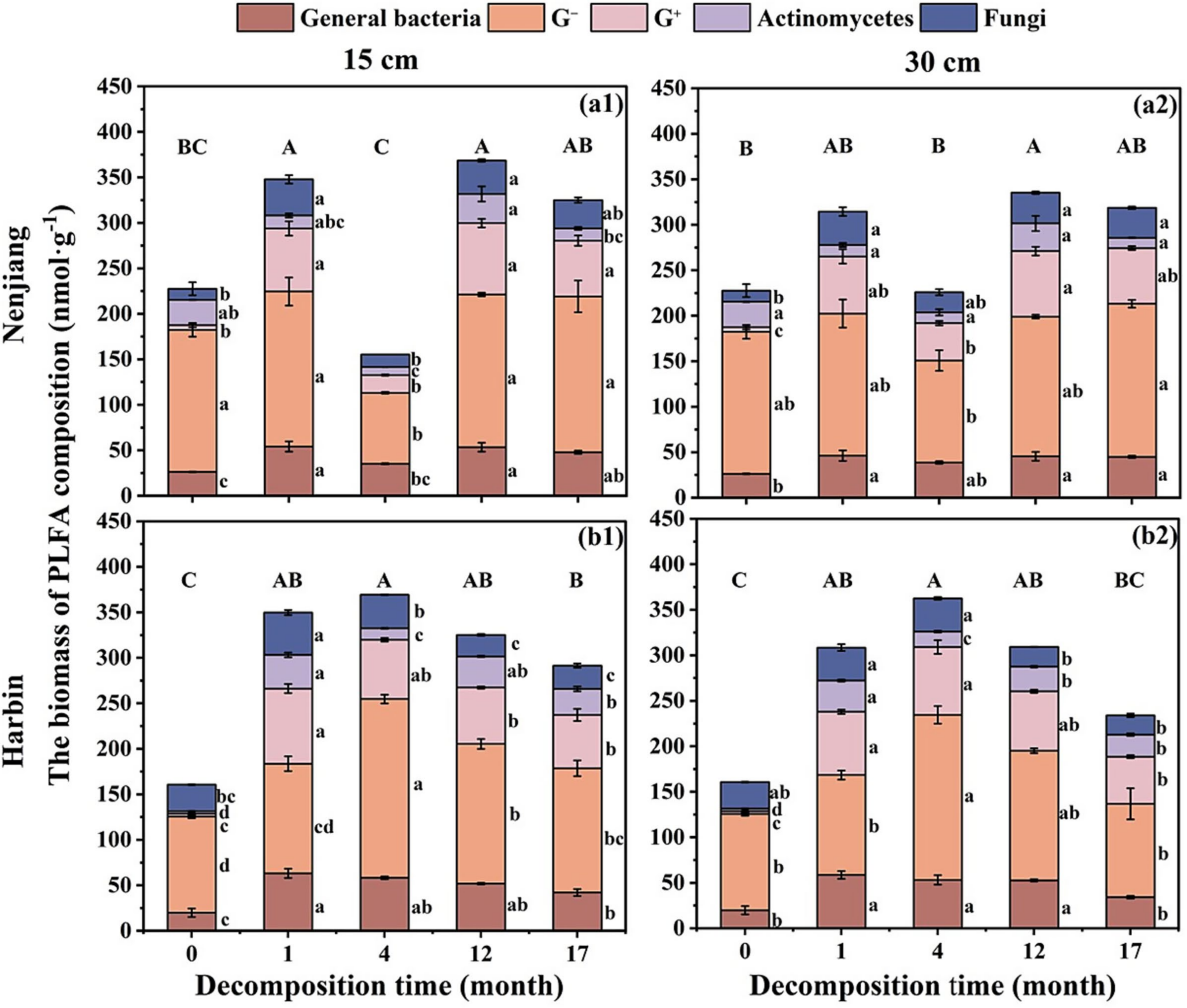
Temporal dynamics of soil microbial group biomass across soil depths at two sites (Nenjiang and Harbin) over successive stages of decomposition at 15 cm and 30 cm burial depths in Nenjiang (a1, a2) and Harbin (b1, b2). GB, General bacteria; G^−^, Gram-negative bacteria; G^+^, Gram-positive bacteria; Act, Actinomycetes. Different lowercase letters indicate significant differences (*p* < 0.05) among decomposition stages within each microbial group at a given depth and site. Uppercase letters indicate significant differences (*p* < 0.05) in total PLFA biomass among stages at a given depth and site.

The microbial activity and the microbial community structure between the two farmland soils differed distinctly (Figure 3; Supplementary Table S5). During the 17-month decomposition stage, the biomass of all microbial groups responded more strongly to decomposition time (T) and site (S) than to soil depth (D), and the S × T interaction was consistently significant (*p* < 0.001; Supplementary Table S5). At the Nenjiang site, the biomass of total PLFAs, general bacteria, G^−^ bacteria and fungi all increased significantly in the first 1 month and then decreased, and rebounded at 12 months, the values reached a maximum and again decreased; At the end of straw decomposition, the values remained significantly higher than that in original soil. At the Harbin site, the biomass of total PLFAs, general bacteria, G^−^ and G^+^ bacteria initially increased over the first 4 months, subsequently declined, yet remained elevated relative to baseline soil levels. The biomass of fungi and actinomycetes increased slightly and decreased to values similar to those in initial soil. The G^+^/G^−^ radio peaked on 1 month at Harbin, and 12 months of decomposition at Nenjiang site, and were all minimal in original soil. The G^−^ biomass was dominant in PLFAs during the decomposition. The G^+^ biomass in Harbin soil was significantly higher than in Nenjiang (66.2 vs. 58.4 nmol g^−1^, *p* = 0.002), and the G^+^ to G^−^ ratio was 26.3% higher (0.48 vs. 0.38) during decomposition stage. Overall, soil microbial communities and indicators of soil microbial community (G^+^/G^−^) were more responsive to site, decomposition time and their interaction, but no effect by soil depth (Supplementary Table S5).

### 3.3 Drivers of soil microbial community composition

**Figure 4:**
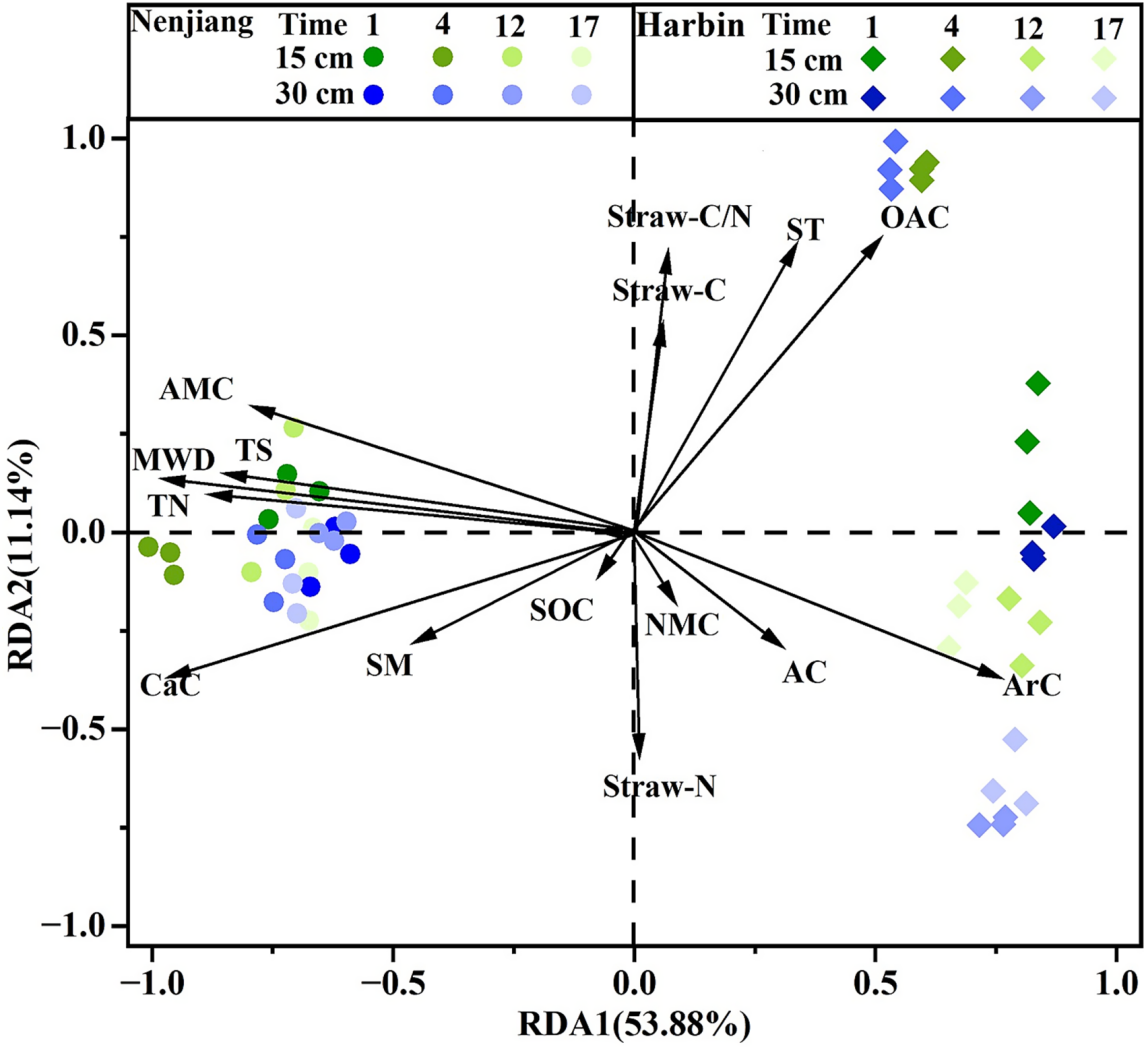
Redundancy analysis (RDA) was used to relate soil microbial community composition (PLFAs) to environmental variables during straw decomposition at two soil depths (15 and 30 cm) in two sites (Nenjiang and Harbin). ST, soil temperature; SM, soil moisture; SOC, soil organic carbon; MWD, mean weight diameter; TS, aggregate tensile strength; AC, Alkyl C; NMC, N-alkyl/methoxyl C; OAC, O-alkyl C; AMC, Anomeric C; ArC, Aryl C; CaC, Carbonyl C.

Redundancy analysis (RDA) was used to evaluate the effects of soil environmental factors, soil properties, and straw chemistry on microbial community composition (35 PLFAs) across different sampling times (1, 4, 12, and 17 months) and depths (15 cm and 30 cm) in Nenjiang and Harbin sites (Figure 4). The environmental variables explained 65.02% of the total variance in the microbial community composition. Soil temperature (ST), Straw-C, Straw-N, Straw-C/N, O-alkyl C (OAC) and Aryl C (ArC) showed strong positive correlations with RDA1, whereas soil moisture (SM), tensile strength (TS), mean weight diameter (MWD), Anomeric C (AMC), and Carbonyl C (CaC) demonstrated notable negative correlations with RDA1, suggesting these variables significant influence on changes in the structure of microbial communities over time. In contrast, SOC and N-alkyl/methoxyl C (NMC) showed no significant correlations (*p* > 0.05). RDA1 (explaining 53.88% of the variation) clearly separated the samples, indicating that the microbial community dynamics driving straw decomposition followed distinct trajectories at the Nenjiang and Harbin sites. The sample points in Harbin were predominantly shaped by the straw chemistry and ST, whereas those in Nenjiang were primarily influenced by soil properties. RDA2 (accounting for 11.14% of the variation) mainly reflects the effects of temporal dynamics and depth. Within a single experimental site, the 15 cm and 30 cm points were basically overlapping. This implies that the effect of burial depth is much less significant than the site effect in regulating straw decomposition processes.

**Figure 5:**
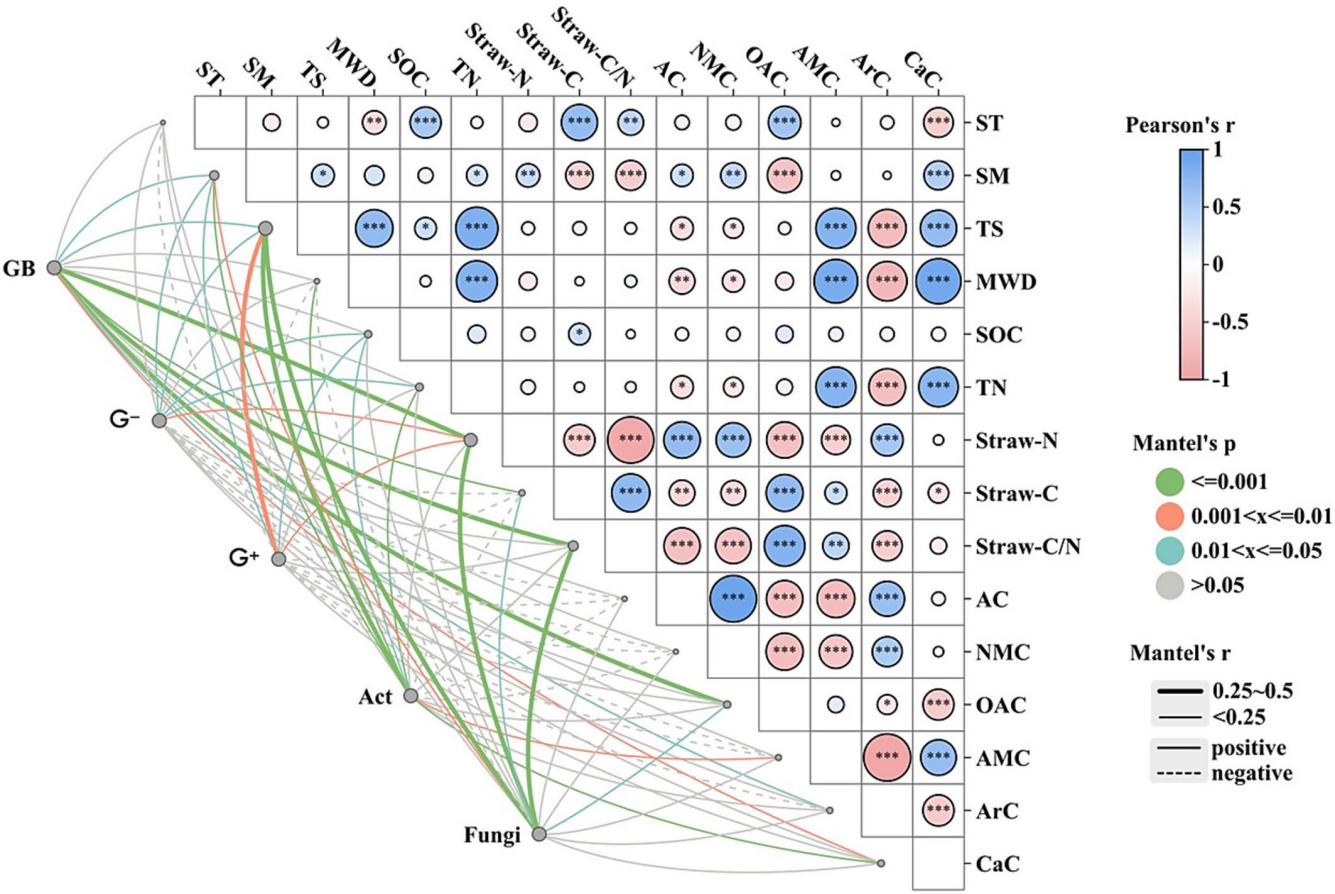
Mantel test between soil microbial groups and environmental variables during straw decomposition. ST, soil temperature; SM, soil moisture; SOC, soil organic carbon; MWD, mean weight diameter; TS, aggregate tensile strength; AC, Alkyl C; NMC, N-alkyl/methoxyl C; OAC, O-alkyl C; AMC, Anomeric C; ArC, Aryl C; CaC, Carbonyl C; GB, General bacteria; G^−^, Gram-negative bacteria; G^+^, Gram-positive bacteria; Act, Actinomycetes. The significance level for each effect is denoted as * (*p* < 0.05), ** (*p* < 0.01), and *** (*p* < 0.001).

The Mantel tests offered additional support for the RDA results, indicating that soil microbial groups had the closest associations with soil environmental factors, soil properties, and straw chemistry (Figure 5). Both general bacteria (GB) and fungi displayed robust positive links with straw-N, straw-C/N, and O-alkyl C (OAC) (*p* = 0.001), along with relatively weak connections to soil moisture (SM) and tensile strength (TS) (*p* < 0.05). Gram-negative bacteria (G^−^) were notably affected by TS (*r* = 0.193, *p* = 0.011), Straw-N (*r* = 0.233, *p* = 0.002), and SM (*r* = 0.113, *p* = 0.037). Gram-positive bacteria (G^+^) showed significant positive correlations with TS (*r* = 0.347, *p* = 0.003), SOC (*r* = 0.215, *p* = 0.017), Straw-N (*r* = 0.244, *p* = 0.008), and SM (*r* = 0.183, *p* = 0.015). Actinomycetes (Act) exhibited strong positive correlations with SM (*r* = 0.209), TS (*r* = 0.478), MWD (*r* = 0.200), TN (*r* = 0.222), and Carbonyl C (CaC, *r* = 0.238), and weaker links with SOC (*r* = 0.126) and Anomeric C (AMC, *r* = 0.156). Relevant network analysis revealed strong positive correlations among soil physical properties (e.g., TS and MWD) and among straw chemical parameters (e.g., Straw-N and Straw-C). Meanwhile, soil moisture (SM) demonstrated negative correlations with several other parameters.

### 3.4 Factors affecting straw remaining mass

**Figure 6:**
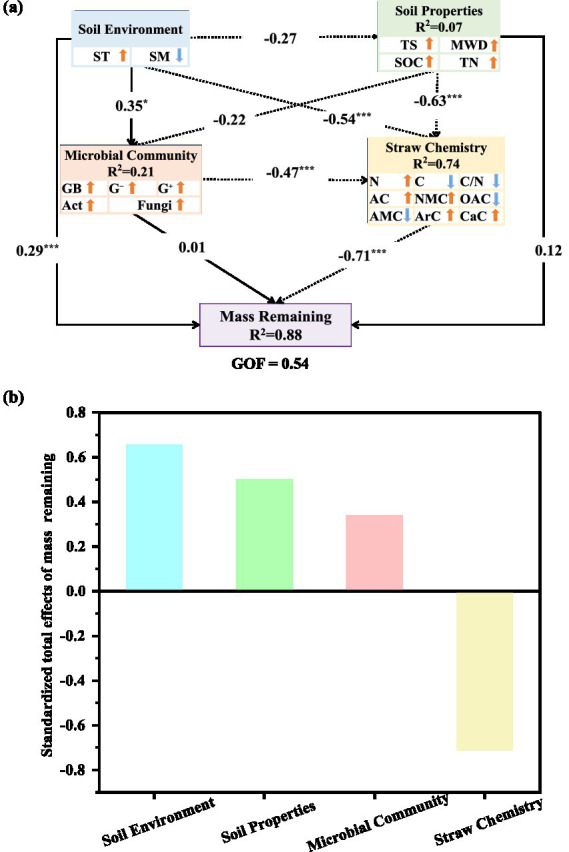
Partial least squares path modeling (PLS-PM) showing the direct and indirect effects of soil environment, soil properties, microbial community composition, and straw chemistry on straw mass remaining during decomposition at two experimental sites (Nenjiang and Harbin) **(a)** and their total standardized effects on straw mass remaining **(b)**. The significance level for each effect is denoted as * (*p* < 0.05), ** (*p* < 0.01), and *** (*p* < 0.001).

Partial least squares path modeling (PLS-PM) was employed to clarify the direct and indirect pathways through which soil environmental variables, soil properties, microbial groups, and straw chemistry modulate straw mass remaining. The integrated model explained 88% of the variation in straw mass remaining during decomposition (Figure 6a). Straw chemistry was the dominant driver (path coefficient = −0.71, *p* < 0.001), contributing substantially to the variation in straw mass remaining (*R*^2^ = 0.74). Soil temperature (ST) and soil moisture (SM) served as key environmental factors that exerted a direct positive effect on straw mass remaining (path coefficient = 0.29, *p* < 0.001). Soil properties exhibited a significant direct negative effect on straw chemistry (path coefficient = −0.63, *p* < 0.001). Notably, the soil environment had a positive direct effect (path coefficient = 0.35, *p* < 0.05) but a negative indirect effect (path coefficient = 0.54, *p* < 0.001) on straw chemistry. As shown in Figure 6b, the standardized total effects revealed that straw chemistry had the most pronounced negative impact on mass remaining, followed by soil properties and the microbial community, whereas the soil environment exhibited a positive overall effect. This suggests that straw chemistry was a critical determinant of decomposition rates, with soil properties and microbial communities playing significant mediating roles.

## 4 Discussion

### 4.1 Response of straw decomposition to soil depth, sites and decomposition time

Early-stage decomposition (0–4 months) accounted for 81.73–84.40% of total maize straw mass loss, with subsequent rates declining toward an asymptotic plateau by month 12 (Figures 1a,b). This result aligns with widely reported “rapid–slow–stable” three-phase model of litter decay (Han et al., 2020; Parsons et al., 2025). Straw mass remaining was site-dependent, with Nenjiang consistently exhibiting higher residual biomass than Harbin throughout decomposition process (Supplementary Table S3; Figures 1a,b). The faster decomposition at Harbin likely reflects warmer average temperatures as a key climatic driver (Supplementary Table S4), although concurrent differences in soil texture, moisture regimes, and baseline microbial communities may also contribute to the site effect (Angst et al., 2021; Chang et al., 2026). Within the tested shallow depth range (15–30 cm), decomposition dynamics did not differ significantly between soil depths (*p* = 0.618). The lack of a depth effect within the 15–30 cm range agrees with incubation studies reporting similar decomposition rates across depths (Kanal, 1995), but contrasts with field observations where deeper subsurface layers often show slower decay due to reduced O₂ and nutrient availability (Pommeresche et al., 2006; Yu et al., 2025). This discrepancy may be due to the depletion of readily decomposable organic matter, hemicellulose, and cellulose within the 17-month decomposition period, leaving insufficient substrate to reveal a significant depth effect (Solly et al., 2014; Yu et al., 2025; Zhao et al., 2025). Notably, the 15 cm and 30 cm depths likely represent a relatively narrow environmental gradient, as both layers remain within the plough layer where O₂ diffusion, temperature fluctuations, and microbial biomass are comparatively favorable for decomposition. Consequently, the absence of a depth effect should not be interpreted as evidence that burial depth is universally unimportant, but rather that it may be negligible within the shallow range typical of agricultural straw-return practices.

Solid-state ^13^C-NMR spectroscopy revealed chemical composition of maize residues underwent dynamic changes, manifesting as progressive O-alkyl C depletion concomitant with Alkyl C and Aryl C enrichment (Figures 1c–f). This pattern aligns with the mass remaining of straw, whereby microbial communities sequentially exploit substrates of decreasing energetic yield, leaving behind progressively more recalcitrant residues (Bao et al., 2020). The pronounced decline in O-alkyl C from 51% to ~37% over 17 months reflected the rapid mineralization of polysaccharides, which constitute the primary energy sources for decomposer microorganisms (Kögel-Knabner, 2002). Alkyl C accumulation is attributed to the recalcitrant nature of hydrophobic precursors and microbial preference for labile polysaccharides, whereas Aryl C enrichment indicates selective preservation of lignin derivatives (Lorenz et al., 2007). The trajectory of this structural convergence toward humus-like material was strongly site-dependent yet depth-invariant, implicating climatic (site) drivers over burial effects in regulating straw humification dynamics (Wang et al., 2012; Berenstecher et al., 2021). The Alkyl C/O-alkyl C ratio and aromaticity surged at both sites but remained higher at Harbin (Figure 2), indicating more advanced decomposition.

Using a single maize straw type isolated burial depth and microclimate effects from substrate confounding. Straw chemistry is thus treated as a dynamic feedback variable—both responding to and directing microbial succession—rather than a static initial condition. While climatic and edaphic effects remain entangled, the site-dependence of straw chemistry and microbial communities (Sections 4.2 and 4.3) points to temperature, soil properties, and indigenous microbial backgrounds as interacting co-drivers.

### 4.2 Dynamics of soil microbial communities and controlling mechanisms during straw decomposition

The total microbial biomass, general bacteria, Gram-negative (G^−^) bacteria, and fungi increased significantly during the early decomposition phase (1–4 months), due to the rapid colonization of straw-derived labile carbon substrates by r-selected microbial populations (Li et al., 2020; Guo et al., 2025). This finding is consistent with classical decomposition theory predicting that copiotrophic populations, equipped with rapid proliferation and high catabolic activity, dominate early decomposition stages when nutrient accessibility is maximal (Kramer and Gleixner, 2006; Yang et al., 2023). Following the initial biomass maximum, progressive microbial decline indicated a shift toward recalcitrant substrate metabolism, consistent with carbon limitation dynamics in temperate farmland (Zhang et al., 2025b). Nenjiang exhibited a distinct biphasic trajectory characterized by biomass recovery at month 12 following an initial decline at month 4, diverging from the pattern observed at Harbin; this asynchronous response likely arises from substrate availability (driven by intermediate decomposition products or seasonal environmental fluctuations) and reflects spatial heterogeneity in thermal regimes, and edaphic properties detailed in Section 4.1 (Devêvre and Horwáth, 2000; Sun et al., 2018; Briones et al., 2025). At the end of the 17-month decomposition, total PLFAs, general bacteria, Gram-negative bacteria, and fungi remained elevated above initial soil levels, indicating a lasting legacy effect of straw addition on microbial carrying capacity (Li et al., 2025; Zhang et al., 2025a).

The G^+^/G^−^ ratio peaked at 1 month in Harbin compared with 12 months in Nenjiang, with Harbin exhibiting significantly greater G^+^ biomass and a higher G^+^/G^−^ ratio (Supplementary Figure S2). These discrepancies mirror the site-specific differences in soil properties and climate described in Section 4.1, which directly affect microbial metabolic activity and community composition (Angst et al., 2021; Chang et al., 2026). Statistical results further clarified that soil microbial community was affected by site, decomposition time and their interaction, but not by soil depth within the 15–30 cm range. This result is consistent with the depth-invariant straw mass loss reported above (Wang et al., 2012; Angst et al., 2021).

Redundancy analysis (RDA) and Mantel tests identified environmental variables as primary drivers of soil microbial community structure. Microbial communities at the warmer Harbin site exhibited heightened responsiveness to labile organic matter inputs, as reflected in their tight coupling with readily available carbon and straw nitrogen. Conversely, communities at the colder, more fertile Nenjiang site were governed primarily by inherent soil properties rather than straw-derived nutrient pools (Murase et al., 2012; Ma et al., 2025; Yuan et al., 2025). Close associations between specific microbial groups and soil structural properties (particularly strong correlations of Actinobacteria with tensile strength and mean weight diameter, and of Gram-negative and Gram-positive bacteria with tensile strength) underscore the role of soil architecture in shaping microbial ecological niches. These findings support the notion that soil structure modulates microbial access to substrates and oxygen, thereby governing straw decomposition rates (Kravchenko et al., 2019).

### 4.3 Statistical predictors of straw decomposition

As conceptualized in Section 4.1, straw chemistry in this study represents a dynamic feedback variable. PLS-PM analysis revealed straw chemistry was the strongest statistical predictor of straw mass remaining in the cold, high-latitude regions. Specifically, the progressive shift from labile to recalcitrant C fractions, evidenced by the relative increases in N content, Alkyl C (AC), N-alkyl/methoxyl C (NMC), Aryl C (ArC), and Carbonyl C (CaC), as well as the decrease of C content, C/N ratio, O-alkyl C (OAC) and Anomeric C (AMC), was significantly linked to faster straw decomposition and lower mass remaining. These changes support the interpretive hypothesis that straw chemistry is strongly associated with decomposition of straw (Deng et al., 2010; Prescott, 2010).

Soil environment explained substantial variance in straw mass remaining (path coefficient: 0.65), with the temperature–moisture interaction accounted for additional variance (path coefficient: 0.29). Although soil temperature increases are generally regarded as accelerating decomposition (Devêvre and Horwáth, 2000), our findings demonstrate the temperature effect is highly dependent on moisture. Warming temperature coupled with drought may induce microbial water stress and suppress extracellular enzyme activity, thereby statistically coinciding with constraining decomposition (Ma et al., 2025). Soil properties and microbial communities exerted showed no direct statistical association with straw mass remaining, yet both variables were linked to mass remaining through their covariance with mass remaining indirectly via straw chemistry (path coefficients: −0.63 and −0.47), underscoring the importance of biological drivers in straw chemical transformation (Bao et al., 2020).

### 4.4 Limitations and future considerations

It should be noted that the two sites differed in multiple environmental attributes beyond temperature, including soil texture, pH, nutrient availability, and indigenous microbial communities. Because these factors were not manipulated independently in the present study, their relative contributions cannot be quantitatively disentangled. Consequently, temperature is interpreted here as a major but not exclusive driver of the observed site differences. Furthermore, because only two relatively shallow burial depths (15 and 30 cm) were examined, the null depth effect reported here should not be extrapolated to deeper soil profiles. Significant depth-induced constraints on decomposition (mediated by O₂ limitation, thermal damping, and reduced microbial biomass) may well emerge at greater depths (Pommeresche et al., 2006; Yu et al., 2025) Cross-site transplant experiments or controlled-temperature incubations would be required to rigorously isolate the specific effects of thermal regimes from edaphic and biotic confounders and future studies incorporating a broader depth gradient (e.g., 0, 15, 30, and ≥50 cm) are needed to identify the critical threshold at which burial depth becomes a limiting factor for straw decomposition. The PLS-PM model should be viewed as an exploratory tool for generating mechanistic hypotheses that require subsequent experimental testing—such as reciprocal transplant experiments, isotope-tracking studies, or controlled microcosm manipulations—to establish definitive causal relationships.

## 5 Conclusion

This 17-month field study demonstrated that straw mass remaining was governed primarily by site and decomposition time, with no significant effect of soil depth. The depletion of labile carbohydrates (O-alkyl C) and the concurrent enrichment of recalcitrant fractions (Alkyl C and Aryl C) indicated progressive straw chemical transformation. PLS-PM analysis identified straw chemistry as the dominant direct driver of straw mass remaining, whereas soil properties and microbial communities exerted indirect effects via changes in straw chemical composition. Specific microbial taxa were significantly correlated with straw chemistry (e.g., bacteria and fungi with straw-N, C/N ratio, and O-alkyl C; Actinomycetes with Carbonyl C), suggesting coupled straw chemistry–microbial dynamics. These findings highlight the importance of straw chemical evolution in regulating decomposition in cold-region Mollisols.

## Data Availability

The original contributions presented in the study are included in the article/Supplementary material, further inquiries can be directed to the corresponding author.
