# Supplementary material for: Straw chemistry overrides microbial and environmental controls on straw decomposition in high-latitude mollisols
Source: Front Microbiol. 2026 Jun 23;17:1859074. doi: 10.3389/fmicb.2026.1859074 (PMC13337632; doi:10.3389/fmicb.2026.1859074)
Supplement: Supplementary file 1 [file Data_Sheet_1.docx]

**Supplementary information**

# **Straw Chemistry Overrides Microbial and Environmental Controls on Straw Decomposition in High-Latitude Mollisol**

**Ya Han^1^, Xianghai Meng^2^†, Heng Jiang^3^, Longkai Yue^4^, Ziyi Wang^1^, Qingqing Chen^1^, Shuihong Yao^5^***

^1^ Hebei Province Key Laboratory of Sustained Utilization and Development of Water Resources, Hebei GEO University, Shijiazhuang, 052161, China

^2^ Key Lab of Crop Drought Tolerance Research of Hebei Province, Institute of Dry Farming, Hebei Academy of Agriculture and Forestry Sciences, Hengshui, 053000, China

^3^ National-Regional Joint Engineering Research Center for Soil Pollution Control and Remediation in South China, Institute of Eco-Environmental and Soil Sciences, Guangdong Academy of Sciences, Guangzhou, 510650, China

^4^ Information Center of Ministry of Ecology and Environment, Beijing, 100029, China

^5^ State Key Laboratory of Efficient Utilization of Arable Land in China, the Institute of Agricultural Resources and Regional Planning, Chinese Academy of Agricultural Sciences, Beijing, 100081, China

*** Correspondence:**

Shuihong Yao: [Yaoshuihong@caas.cn](mailto:Yaoshuihong@caas.cn)

†These authors have contributed equally to this work

Table S1 Basic soil physical and chemical properties before the experiment at Nenjiang and Harbin.

|  | Experimental site | |
| --- | --- | --- |
|  | Nenjiang | Harbin |
| pH | 6.0±0.1 b | 6.9 ± 0.1 a |
| Soil organic C (g·kg^-1^) | 19.7± 0.2 a | 19.4 ± 0.2 a |
| TN (g·kg^-1^) | 1.8± 0.1 a | 1.5 ± 0.1 b |
| TP (g·kg^-1^) | 0.4± 0.1 a | 0.3 ± 0.1 a |
| TK (g·kg^-1^) | 20.6± 0.5 b | 24.8 ± 1.4 a |
| Available phosphorus (mg·kg^-1^) | 67.2± 1.8 a | 43.2± 1.6 b |
| Available potassium (mg·kg^-1^) | 307.8± 1.6 a | 152.4± 0.7 b |
| Clay (%) | 29 | 27 |
| Silt (%) | 38 | 43 |
| Sand (%) | 33 | 30 |

Note: TN, Soil total nitrogen; TP, Soil total phosphorus Different lowercase letters in a column indicate significant differences between the two soils using t test at *p* < 0.05 level (mean ± SD, n = 3).

Table S2 The classification standard of organic functional groups of maize straw.

| Chemical shift (ppm) | Functional group | Specific functional groups | Representative compounds | References |
| --- | --- | --- | --- | --- |
| 0–44 | Alkyl C  (AC) | CH_3_,(CH_2_)_n_ | Lipids, hemicellulose, proteins, aliphatic biopolymers such as cutin and suberin | Jokic et al., 2003  Kogel-Knabner, 2002  Wang et al.,2012  Zak et al.,2017 |
| 44–68 | N-alkyl/methoxyl C  (NMC) | NCH, OCH_3_ | Lignin, polypeptides |  |
| 68–94 | O-alkyl C  (OAC) | OCH, OC_q_ | Carbohydrates, celluloses, hemicelluloses, polymeric carbohydrates alcohols, ether-bonded aliphatic C |  |
| 94–113 | Anomeric C  (AMC) | O-CH-O  O-C_q_-O | Polysaccharides and ketals |  |
| 113–162 | Aryl C  (ArC) | Aromatic C-H  Aromatic C-C  Aromatic C-O | Lignin-derived phenols such as syringyl, guaiacyl and p-hydroxyphenyl structures |  |
| 162–220 | Carbonyl C  (CaC) | COO/NC=O &RC（=O）R' | Carboxylic acid, amide, poly-peptides, aldehyde,  ketonic and quinone |  |
| (0-44)/(68-94) | Alky C / O-alkyl C | The degree of decomposition of straw was evaluated based on the relative proportion of different functional groups | | Solomon et al., 2007; An et al. 2021; Chen et al. 2024 |
| (113–162)/(0–44+44–68+68–113+113-162+162-220) | Aromaticity |  |  |  |

Table S3 Analysis of variance (ANOVA) for Straw-C, Straw-N content and Straw-C/N

ratio.

| Variables | Straw-C  (g· kg^-1^) | Straw-N  (g· kg^-1^) | Straw-C/N | Mass Remaining（%） |
| --- | --- | --- | --- | --- |
| **Site (S)** |  |  |  |  |
| Nenjiang | 370.20±5.64 a | 12.08±0.19 a | 32.77±1.64 a | 27.72±1.27 a |
| Harbin | 372.98±5.53 a | 12.56±0.19 b | 32.53±2.44 a | 19.54±1.27 b |
| **Depth (D)** |  |  |  |  |
| 15 cm | 366.55±5.43 a | 12.28±0.61 a | 32.17±2.20 a | 26.81±5.09 a |
| 30 cm | 376.63±5.56 a | 12.08±0.56 a | 33.13±1.95 a | 27.72±4.84 a |
| **Time (T)** |  |  |  |  |
| 1 month | 402.76±5.71 a | 8.57±0.61 c | 47.70±3.65 a | 66.37±2.12 a |
| 4 months | 379.83±8.45 b | 13.57±0.97 ab | 28.41±2.16 bc | 17.27±2.12 b |
| 12 months | 362.63±9.23 c | 12.64±0.84 b | 29.08±2.28 b | 16.48±1.78 b |
| 17 months | 341.14±10.68 d | 13.94±1.64 a | 25.41±3.05 c | 8.94±2.30 c |
| ANOVA |  |  |  |  |
| *P* value (S) | 0.511 | **0.032*** | 0.793 | **0.000***** |
| *P* value (D) | **0.022*** | 0.547 | 0.291 | 0.618 |
| *P* value (T) | **0.000***** | **0.000********* | **0.000***** | **0.000***** |
| *P* value (S×D) | 0.068 | 0.085 | **0.005**** | 0.713 |
| *P* value (S×T) | 0.430 | **0.000***** | **0.000***** | 0.123 |
| P value (D×T) | 0.988 | 0.335 | 0.621 | 0.845 |
| *P* value (S×D×T) | 0.443 | 0.349 | **0.037*** | 0.829 |

Note: The different lowercase letters among treatments indicate significant differences. Significance levels of each effect are marked with * at *p* < 0.05, ** at *p* < 0.01 and *** at *p* < 0.001.

Table S4 Effects of experimental sites (S), soil depth (D) and decomposition time (T) on soil physicochemical properties.

| Variables | ST  (℃) | SM  (%) | SOC  (g·kg^-1^)) | TN  (g·kg^-1^) | MWD  (mm) | TS  (kPa) |
| --- | --- | --- | --- | --- | --- | --- |
| **Site (S)** |  |  |  |  |  |  |
| Nenjiang | 11.47±0.86 b | 24.88±1.12 a | 19.48±0.29 a | 1.50±0.01 a | 0.99±0.03 a | 706.72±46.00 a |
| Harbin | 14.18±0.81 a | 20.95±0.69 b | 19.48±0.13 a | 1.31±0.01 b | 0.30±0.01 b | 252.17±17.43 b |
| **Depth (D)** |  |  |  |  |  |  |
| 15 cm | 13.06±0.90 a | 23.14±0.95 a | 19.50±0.24 a | 1.41±0.02 a | 0.65±0.08 a | 463.78±53.79 a |
| 30 cm | 12.59±0.86 a | 22.69±1.07 a | 19.46±0.22 a | 1.40±0.03 a | 0.64±0.07 a | 495.11±23.76 a |
| **Time (T)** |  |  |  |  |  |  |
| 1 month | 16.09±0.58 b | 17.22±0.81 c | 19.94±0.27 a | 1.42±0.03 a | 0.72±0.12 a | 530.65±72.98 b |
| 4 months | 17.30±0.27 a | 26.83±0.75 a | 20.30±0.26 a | 1.44±0.04 a | 0.54±0.09 b | 637.27±99.20 a |
| 12 months | 9.77±0.38 c | 23.32±1.10 b | 18.93±0.19 b | 1.38±0.02 a | 0.64±0.10 a | 300.45±44.07 c |
| 17 months | 8.15±0.58 d | 24.29±1.28 ab | 18.75±0.30 b | 1.39±0.03 a | 0.68±0.12 a | 449.41±79.35 b |
| ANOVA |  |  |  |  |  |  |
| *P* value (S) | **0.000***** | **0.000***** | 0.961 | **0.000***** | **0.000***** | **0.000***** |
| *P* value (D) | **0.000***** | 0.505 | 0.858 | 0.893 | 0.865 | 0.245 |
| *P* value (T) | **0.000********* | **0.000***** | **0.000***** | 0.083 | **0.000***** | **0.000***** |
| *P* value (S×D) | **0.001**** | 0.558 | 0.078 | 0.367 | 0.176 | **0.026*** |
| *P* value (S×T) | **0.000***** | **0.002**** | **0.000***** | 0.222 | **0.002**** | **0.001**** |
| P value (D×T) | **0.000***** | 0.593 | 0.303 | 0.972 | 0.500 | 0.332 |
| *P* value (S×D×T) | **0.000***** | **0.017*** | 0.714 | 0.509 | 0.149 | 0.076 |

Note: ST, Soil temperature; SM, Soil moisture; SOC, Soil organic carbon; TN, Soil total nitrogen; MWD, Mean weight diameter; TS, Aggregate tensile strength. The different lowercase letters among treatments indicate significant differences. Significance levels of each effect are marked with ^*^ at *p* < 0.05, ** at *p* < 0.01 and *** at *p* < 0.001.

Table S5 Effects of experimental site (S), soil depth (D), and decomposition time (T) on the biomass of different microbial groups, and the Gram-positive-to-Gram-negative bacteria ratio

| Variables | Total PLFAs  (nmol·g^-1^) | GB  (nmol·g^-1^) | G⁻  (nmol·g^-1^) | G⁺  (nmol·g^-1^) | Fungi  (nmol·g^-1^) | Act  (nmol·g^-1^) | ‌G⁺/G⁻ |
| --- | --- | --- | --- | --- | --- | --- | --- |
| **Site (S)** |  |  |  |  |  |  |  |
| Nenjiang | 299.99±15.50 a | 45.85±1.68 b | 147.91±7.34 a | 58.39±3.98 b | 30.97±1.88 a | 16.86±2.21 b | 0.38±0.01 b |
| Harbin | 318.75±10.14 a | 51.71±2.10 a | 142.93±6.97 a | 66.17±2.24 a | 31.08±1.90 a | 26.86±1.75 a | 0.48±0.02 a |
| **Depth (D)** |  |  |  |  |  |  |  |
| 15 cm | 316.57±15.09 a | 50.71±2.03 a | 149.34±7.76 a | 62.06±4.01 a | 31.81±2.15 a | 22.65±2.44 a | 0.42±0.03 a |
| 30 cm | 302.16±10.89 a | 46.85±1.87 a | 141.49±6.44 a | 62.50±2.47 a | 30.24±1.58 a | 21.07±2.03 a | 0.45±0.02 a |
| **Time (T)** |  |  |  |  |  |  |  |
| 1 month | 330.06±13.46 ab | 55.47±2.93 a | 139.25±9.06 a | 71.04±3.42 a | 39.74±2.11 a | 24.57±3.49 ab | 0.53±0.04 a |
| 4 months | 280.53±28.19 c | 46.60±3.07 bc | 143.34±15.04 a | 50.45±6.66 b | 27.58±3.05 b | 12.56±1.16 c | 0.34±0.02 c |
| 12 months | 334.61±9.12 a | 50.81±1.79 ab | 154.40±3.08 a | 69.43±2.41 a | 29.04±1.99 b | 30.92±2.64 a | 0.45±0.01 b |
| 17 months | 292.26±14.45 bc | 42.24±1.82 c | 144.69±10.09 a | 58.20±2.25 b | 27.74±1.69 b | 19.39±2.30 b | 0.42±0.02 b |
| ANOVA |  |  |  |  |  |  |  |
| *P* value (S) | 0.092 | **0.003**** | 0.338 | **0.002**** | 0.926 | **0.000***** | **0.000***** |
| *P* value (D) | 0.191 | **0.043*** | 0.135 | 0.853 | 0.227 | 0.359 | **0.002**** |
| *P* value (T) | **0.002**** | **0.000***** | 0.215 | **0.000***** | **0.000***** | **0.000***** | **0.000********* |
| *P* value (S×D) | 0.149 | 0.860 | 0.064 | 0.359 | 0.041 | 0.573 | 0.595 |
| *P* value (S×T) | **0.000***** | **0.000***** | **0.000***** | **0.000***** | **0.000***** | **0.000***** | **0.000********* |
| *P* value (D×T) | 0.076 | 0.667 | 0.167 | **0.003**** | **0.037** | 0.366 | **0.004**** |
| *P* value (S×D×T) | 0.351 | 0.298 | 0.145 | 0.352 | 0.491 | 0.916 | 0.089 |

Note: PLFAs, The phospholipid fatty acids; GB, General Bacteria; G⁻, Gram-negative bacteria; G⁺, Gram-positive bacteria; Act, Actinomycetes. The different lowercase letters among treatments indicate significant differences. Significance levels of each effect are marked with * at *p* < 0.05, ** at *pp* < 0.01 and *** at *p* < 0.001.


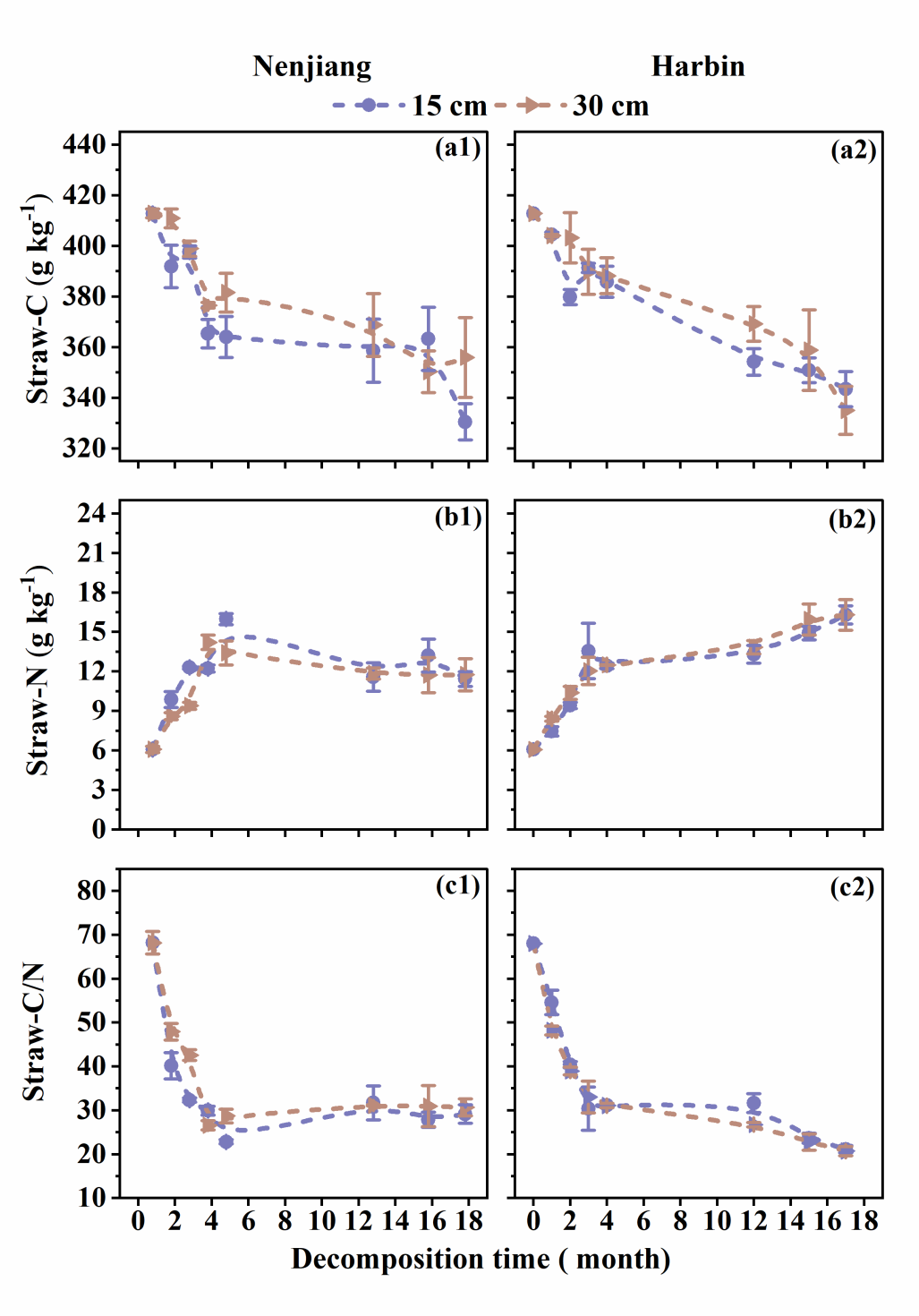


Figure S1 Temporal dynamics of straw C (a1, a2), straw N (b1, b2), and straw C/N (c1, c2) in litterbags incubated at different soil depths (15 and 30 cm) at the Nenjiang and Harbin sites during straw decomposition. Error bars represent the standard error of the mean (n = 3).


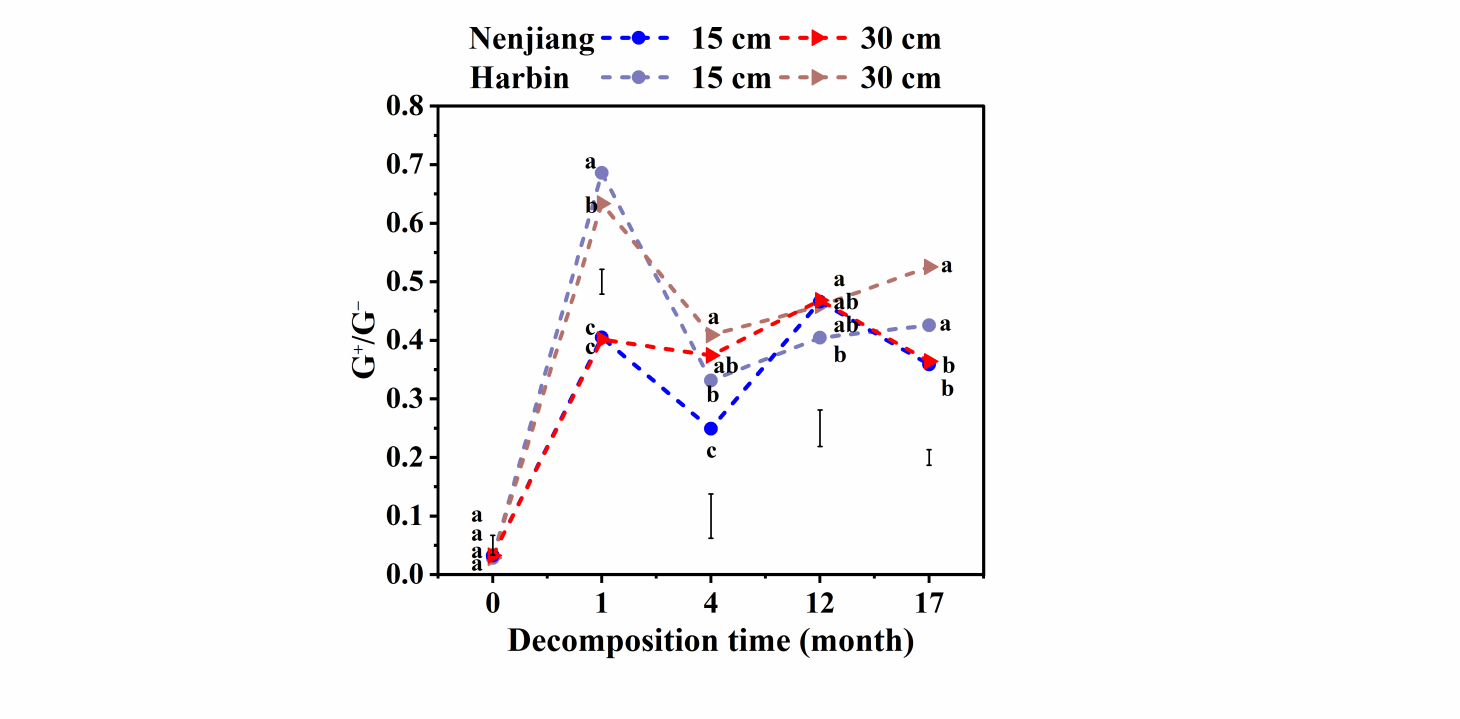


Figure S2 Dynamic changes for the biomass of soil microbial groups in different soil depths at two sites (Nenjiang and Harbin) during different decomposition stages. The different lowercase letter among treatments means significant differences. G⁻, Gram-negative bacteria; G⁺, Gram-positive bacteria.

**References**

An, Z., Bernard, G. M., Ma, Z., Plante, A. F., Michaelis, V. K., Bork, E. W., et al. (2021) Forest land-use increases soil organic carbon quality but not its structural or thermal stability in a hedgerow system. *Agric. Ecosyst. Environ.* 321: 107617. doi: 10.1016/j.agee.2021.107617

Chen, S., Xia, X., Ding, Y., Feng, X., Lin, Q., Li, T., et al. (2024) Changes in aggregate-associated carbon pools and chemical composition of topsoil organic matter following crop residue amendment in forms of straw, manure and biochar in a paddy soil. *Geoderma* 448:116967. doi: 10.1016/j.geoderma.2024.116967

Jokic, A., Cutler, J. N., Ponomarenko, E., Kamp, G. V. D., and Anderson, D. W. (2003) Organic carbon and sulfur compounds in wetland soils: insights on structureand transformation processes using K-edge XANES and NMR spectroscopy. *Geochim. Cosmochim. Ac.* 67, 2585–2597. doi: 10.1016/S0016-7037(03)00283-4

Solomon, D., Lehmann, J., Kinyangi, J., Amelung, W., Lobe, I., Pell, A., et al. (2007). Long-term impacts of anthropogenic perturbations on dynamics and speciation of organic carbon in tropical forest and subtropical grassland ecosystems. *Global Change Biol.* 13, 511–530. doi: 10.1111/j.1365-2486.2006.01304.x

Kögel-Knabner, I. (2002). The macromolecular organic composition in plant and microbial residues as input to soil. *Soil Biol. Biochem.* 34, 139–162. doi: 10.1016/S0038-0717(01)00158-4

Wang, X., Sun, B., Mao, J., Sui, Y., Cao, X. (2012). Structural convergence of maize and wheat straw during two-year decomposition under different climate conditions. *Environ. Sci. Technol.* 46, 7159–7165. doi: 10.1021/es300522x

Zak, D. R., Freedman, Z. B., Upchurch, R. A., Steffens, M., Kgel-Knabne, I. (2017). Anthropogenic N deposition increases soil organic matter accumulation without altering its biochemical composition. *Global Change Biol.* 23. 933–944. doi: 10.1111/gcb.13480
